# Effects of Salinity, Temperature, and Diet on the Biological Characteristics of *Brachionus plicatilis* Müller, 1786

**DOI:** 10.3390/biology14070878

**Published:** 2025-07-18

**Authors:** Quynh-Anh Tran-Nguyen, Truong Nhat Phan, Quang-Anh Tran, Hong Thi Mai, Thao Linh Phan Thi, Dang Doan Phan, Mau Trinh-Dang

**Affiliations:** 1 Faculty of Biology, Agriculture and Environmental Science, The University of Da Nang—University of Science and Education, 459 Ton Duc Thang St., Danang 550000, Vietnam; quynhanhtn20286@gmail.com (Q.-A.T.-N.); nhattruong.wbf@gmail.com (T.N.P.); pddang@gmail.com (D.D.P.); 2 Environment & Biological Resource (DN-EBR), University of Science and Education, Danang 550000, Vietnam; quanhtran1999@gmail.com (Q.-A.T.); maithihongb9@gmail.com (H.T.M.); thaolinhphan99@gmail.com (T.L.P.T.); 3 Institute of Life Science (ILS), 9/621 Vo Nguyen Giap St, Linh Trung Ward, Thu Duc City, Ho Chi Minh City 700000, Vietnam

**Keywords:** rotifer, adaptive strategies, fecundity, lifespan, cultural condition

## Abstract

This study investigates the effects of salinity, temperature, and diet on the biological characteristics of the rotifer *Brachionus plicatilis*, an essential live feed in aquaculture. The results indicate that environmental factors have a significant influence on reproductive traits and survival. At a salinity of 5 ppt, *B. plicatilis* achieved the highest fecundity (25.50 ± 0.58 inds.), while the longest lifespan (273.00 ± 72.52 h) was observed at 35 ppt. The temperature had a strong influence on developmental rates, with the shortest juvenile period recorded at 35 °C (8.00 ± 0.00 h) and the longest lifespan at 20 °C (270.62 ± 30.38 h). The diet also played a critical role, with *Chlorella vulgaris* supporting maximum fecundity, whereas mixed diets prolonged lifespan to 290.50 ± 62.83 (h). These findings provide valuable insights into optimizing rotifer culture systems to improve aquaculture productivity and sustainability.

## Simple Summary

Tiny aquatic animals known as rotifers (*Brachionus plicatilis*) are a crucial food source for young fish and shrimp in aquaculture, a form of fish farming. However, their growth and reproduction can vary significantly depending on their living conditions. This study examined the impact of salt levels in the water, water temperature, and diet on the health and reproductive ability of these organisms. We found that rotifers produced the most offspring in low-salinity water (5 ppt), but lived the longest in saltier water (35 ppt). Warmer temperatures (35 °C) made them grow up faster, while cooler temperatures (20 °C) helped them live longer. Diet also played a role: a specific algae (*Chlorella vulgaris*) boosted offspring numbers, whereas a mixed algae diet helped them live the longest. These findings help fish farmers create the ideal conditions for growing large quantities of healthy rotifers, which in turn makes fish farming more efficient and sustainable, ultimately supporting our food supply.

## 1. Introduction

Rotifers, particularly *Brachionus plicatilis*, play a vital role in aquatic ecosystems, serving as a key link in the food web by transferring energy from primary producers to higher trophic levels. These microscopic zooplankton are widely distributed across freshwater, brackish, and saltwater environments and contribute significantly to the biological productivity of natural water bodies [1,2]. In aquaculture, rotifers are indispensable as live feed for the larval stages of marine fish and crustaceans. Their small size, high nutritional value—including essential amino acids, fatty acids, and proteins—and ease of cultivation make them ideal for meeting the dietary needs of aquatic larvae, especially during critical developmental stages [3,4]. Studies show that *B. plicatilis* can produce a substantial number of offspring per female under optimal conditions, ensuring a reliable supply of live feed for aquaculture systems [5].

Environmental factors such as salinity, temperature, and diet profoundly affect the growth and reproduction of rotifers [6,7,8]. For instance, *B. plicatilis* exhibits optimal reproductive performance within specific salinity ranges, while extreme salinity levels inhibit their survival and fecundity [9]. Temperature also significantly influences their life cycle and metabolic rates, with different strains often showing distinct thermal preferences for optimal development and reproduction [10,11]. Furthermore, adequate food quality, such as that provided by nutritionally rich microalgae, significantly enhances their growth and reproduction, with high feeding rates often observed under favorable nutritional conditions [12,13,14]. Understanding the intricate biological and ecological responses of rotifers to these environmental variables is crucial for optimizing their cultivation in aquaculture systems.

While comprehensive studies have elucidated the general influence of environmental factors on rotifer biology, there remains a need for more localized investigations that account for unique strain-specific or regional adaptations [15,16]. For native strains, such as the one in this study, critical knowledge gaps remain regarding their precise physiological and demographic responses to varying salinity, temperature, and dietary compositions. This lack of specific data is a significant bottleneck for maximizing their productivity and reliability as a live feed. Therefore, this study systematically quantifies the specific biological responses of this native *B. plicatilis* strain across these environmental parameters, providing crucial region-specific insights and refining our understanding of its adaptive plasticity. Such a foundational baseline is essential for optimizing its culture under local aquaculture conditions, enhancing the overall efficiency and sustainability of aquaculture systems.

## 2. Materials and Methods

### 2.1. B. plicatilis Culturing

The large-type *B. plicatilis* strain was obtained from the Laboratory of Plankton, Department of Biology and Environment, University of Science and Education—The University of Danang. The rotifers were maintained under static culture conditions at 25 ± 1 °C, with a light intensity of 1000 lux and a 16:8 h light–dark photoperiod. This lighting condition is maintained throughout all experiments. The cultures were grown in EPA medium, as described by Peltier and Weber (1985) [17], containing 96 mg NaHCO_3_, 60 mg CaSO_4_, 60 mg MgSO_4_, and 4 mg KCl per liter of distilled water. Sterilized seawater was added to adjust the salinity to the desired level, and the pH was maintained between 7.0 and 7.5. The culture medium was refreshed every two days. Rotifers were fed *Chlorella vulgaris* at a concentration of 1 × 10^6^ cells/mL.

*C. vulgaris* was cultured separately in Bold’s Basal Medium (BBM) [18] under the same light–dark cycle until reaching a density of approximately 20 × 10^6^ cells/mL. Algal cultures were harvested by centrifugation at 3000 rpm for 5 min and stored at 4 °C until use. *C. vulgaris* served as the sole food source for all salinity and temperature experiments, excluding the diet-specific trials. The rotifer cultures were maintained over multiple generations under consistent laboratory conditions prior to experimentation. The temperature was regulated using an air conditioning system, and the lighting was controlled via an automated timer system.

### 2.2. Experimental Design

The experiments were designed following the methodology of Yin & Zhao (2008) [6], using a 96-well cell culture tray. Each well contained 1 mL of volume, comprising 0.3 mL of culture medium and one young *B. plicatilis* individual, less than 2 h old. A total of 56 young rotifers were included in the experiments, which tested six salinity treatments (5, 15, 20, 25, 30, and 35 parts per thousand (ppt); four temperature treatments (20 °C, 25 °C, 30 °C, and 35 °C); and four diet treatments (0%, 20%, 50%, and 100% microalgae), with four replicates each. The salinity, temperature, and diet experiments were conducted independently.

Samples were examined every 2 h under a stereomicroscope (Leica S9i, Leica Microsystems, Wetzlar, Germany) to record individual biological characteristics. Observations continued until all rotifers had died. The observed parameters included the juvenile period, embryonic development time, spawning times, spawning interval, fecundity, and lifespan [19]. The juvenile period (h) was defined as the duration from birth until the first appearance of reproductive eggs, indicating the onset of sexual maturity. The embryonic development time (h) refers to the interval between the moment a female begins carrying eggs and the successful hatching of offspring. Spawning times (h) represented the duration (in hours) between the first and the last egg-carrying event during the female’s lifespan. The spawning interval (h) was calculated as the time between two consecutive spawning events. Fecundity (individuals per female) was determined as the total number of neonates produced by an individual female over her entire life. Lastly, the lifespan period (h) was recorded as the total duration from birth to natural death of each individual.

### 2.3. Culture Conditions According to Salinity, Temperature, and Diet

Salinity experiment: To investigate the effects of salinity on *B. plicatilis*, individuals were cultured under six salinity levels: 5, 15, 20, 25, 30, and 35 ppt. All treatments were maintained at a constant temperature of 25 ± 1 °C, pH 7.0. Rotifers were fed exclusively with *Chlorella vulgaris* at a concentration of 1 × 10^6^ cells/mL throughout the experiment.

Temperature experiment: The effects of temperature were examined by culturing *B. plicatilis* at four different temperatures: 20 °C, 25 °C, 30 °C, and 35 °C. Culture trays were placed inside a Memmert incubator (Memmert GmbH + Co. KG, Schwabach, Germany) to ensure precise thermal regulation. Based on the results from the salinity experiment, the salinity was fixed at 5 ppt, with the pH maintained at 7.0. Rotifers were provided with *C. vulgaris* at 1 × 10^6^ cells/mL as the sole food source.

Diet experiment: To assess the impact of dietary composition, rotifers were subjected to four feeding treatments: (1) 100% *Chlorella* (100A), (2) 50% baker’s yeast and 50% *Chlorella* (50Y:50A), (3) 80% baker’s yeast and 20% *Chlorella* (80Y:20A), and (4) 100% baker’s yeast (100Y). Concentrated *C. vulgaris* was diluted to 10 × 10^6^ cells/mL for the 100A treatment, and fresh baker’s yeast was similarly prepared for the 100Y treatment. Mixed diets were formulated by combining 100A and 100Y in ratios of 1:1 and 1:4 to create the 50Y:50A and 80Y:20A treatments, respectively. The concentrations of both *C. vulgaris* and yeast cells were quantified using a counting chamber (0.1 mm, 1/400 mm^2^, Paul Marienfeld GmbH & Co. KG, Lauda-Königshofen, Germany). Environmental conditions for the diet experiment were standardized across treatments: a salinity of 5 ppt, a temperature of 25 ± 1 °C, and a pH of 7.0.

### 2.4. Data Analysis

Descriptive statistics and analysis of variance (ANOVA) were employed to assess differences in the mean values of biological characteristics across treatments. Tukey’s Honest Significant Difference (HSD) test was applied as a post hoc analysis to identify statistically significant pairwise differences between treatment groups. All statistical analyses were conducted using RStudio software (2025.05.1-513) [20].

To visually represent the trade off strategy between the fecundity and lifespan of *B. plicatilis* under various cultural conditions, the average values of each treatment were normalized to 0–1 values using min–max scaling. The normalization formula applied was as follows:

$$X_{normalized}=\frac{X-X_{min}}{X_{max}-X_{min}}$$

*X_normalized_* represents the normalized value, *X* is the original observed value for the characteristic (fecundity or lifespan), and *X_max_* and *X_min_* are the maximum and minimum value of that characteristic across the entire dataset (all treatments combined).

Subsequently, a bidirectional bar chart was generated, with normalized fecundity values plotted in the positive direction and normalized lifespan values plotted in the negative direction.

## 3. Results

### 3.1. Effects of Salinity on B. plicatilis

In general, increasing salinity tends to prolong the juvenile period, embryonic development time, spawning times, spawning interval, and lifespan while reducing fecundity, indicating that salinity significantly influences the biological characteristics of *B. plicatilis*.

**Figure 1:**
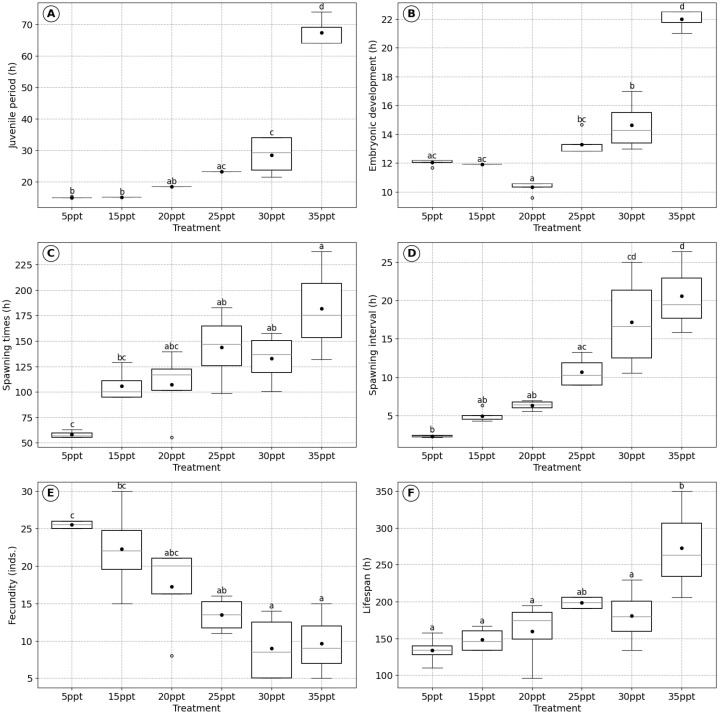
Effect of salinity on the biological characteristics of *B. plicatilis*: (**A**) juvenile period, (**B**) embryonic development time, (**C**) spawning times, (**D**) spawning interval, (**E**) fecundity, and (**F**) lifespan. Data are presented as mean ± standard error based on four replicates. Different lowercase letters above boxplots indicate significant differences among treatments according to Tukey’s HSD post-hoc test (*p* < 0.05).

Specifically, the juvenile period of *B. plicatilis* was shortest at 5 ppt salinity (14.96 ± 0.25 h) and progressively increased with higher salinity, reaching its maximum at 35 ppt (67.33 ± 5.77 h) (Figure 1A). Significant differences were observed among the treatments (*p* < 0.001), with longer juvenile periods recorded at salinities of 25 ppt, 30 ppt, and 35 ppt compared with 5 ppt and 15 ppt (*p* < 0.05) (Figure 1). This indicates that increasing salinity prolongs the juvenile period. 

The embryonic development time initially decreased from 5 ppt (12.04 ± 0.25 h) to 20 ppt (10.33 ± 0.50 h) but increased at salinities above 20 ppt, peaking at 35 ppt (22.00 ± 0.87 h) (Figure 1B). Statistically significant differences were observed among the treatments (*p* < 0.001), with a general trend of longer embryonic development times at higher salinities. The spawning times were shortest at 5 ppt salinity (58.04 ± 3.79 h) and increased with rising salinity, reaching 181.67 ± 53.31 h at 35 ppt (*p* < 0.01) (Figure 1C). The 5 ppt treatment was significantly different from 25 ppt, 30 ppt, and 35 ppt (*p* < 0.05), while no significant differences were observed between 5 ppt and 15 ppt or 20 ppt (*p* > 0.05). This trend demonstrates that higher salinity extends spawning times. The spawning interval was shortest at 5 ppt (2.28 ± 0.14 h) and increased significantly with higher salinities, reaching 20.57 ± 5.36 h at 35 ppt (*p* < 0.001) (Figure 1D).

The lower salinity levels favored higher and more stable fecundity. This parameter was recorded at its highest level at 5 ppt (25.50 ± 0.58 individuals) and decreased significantly with increasing salinity, dropping to 9.00 ± 4.69 individuals at 30 ppt and 9.67 ± 5.03 individuals at 35 ppt (*p* < 0.001) (Figure 1E). In contrast, lifespan positively correlated with salinity and was strongly associated with spawning times (*p* < 0.001, R = 0.74). Lifespan increased from 134.00 ± 19.60 h at 5 ppt to 273.00 ± 72.52 h at 35 ppt, with significant differences observed among the treatments (*p* < 0.01) (Figure 1F). Higher salinities generally resulted in longer lifespans.

### 3.2. Effects of Temperature on B. plicatilis

**Figure 2:**
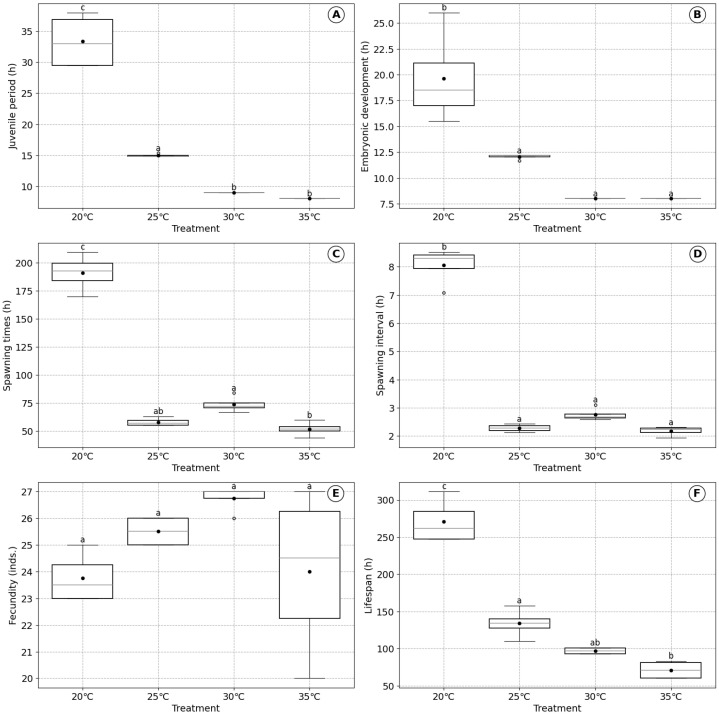
Effect of temperature on the biological characteristics of *B. plicatilis*: (**A**) juvenile period, (**B**) embryonic development time, (**C**) spawning times, (**D**) spawning interval, (**E**) fecundity, and (**F**) lifespan. Data are presented as mean ± standard error based on four replicates. Different lowercase letters above boxplots indicate significant differences among treatments according to Tukey’s HSD post-hoc test (*p* < 0.05).

The significant effects of temperature on the biological characteristics of *B. plicatilis* are shown in Figure 2. The juvenile period of *B. plicatilis* was longest at 20 °C (33.38 ± 4.52 h) and decreased significantly with increasing temperature, reaching its shortest duration within the range of 30–35 °C (*p* < 0.001) (Figure 2A). Similarly, the embryonic development time decreased with increasing temperature (Figure 2B). The embryonic development time was shortest and stabilized at 30 °C and 35 °C (8.00 ± 0.00 h) and was significantly longer at 20 °C (19.62 ± 4.55 h) and 25 °C (12.04 ± 0.25 h) (*p* < 0.001).

The spawning times significantly decreased with rising temperature, from 191.12 ± 16.45 h at 20 °C to 58.04 ± 3.79 h at 25 °C (*p* < 0.001) (Figure 2C). While no significant difference was observed between 30 °C (73.75 ± 7.23 h) and 35 °C (52.00 ± 6.53 h) (*p* > 0.05), the overall trend indicates a substantial reduction in spawning times at temperatures above 25 °C. The spawning interval was longest at 20 °C (8.05 ± 0.66 h) and progressively shortened as temperature increased, reaching 2.17 ± 0.17 h at 35 °C. Significant differences were observed among the treatments (*p* < 0.001). The spawning interval remained short and stable between 25 °C and 35 °C but increased substantially at temperatures below 25 °C (Figure 2D). 

Fecundity was lowest at 20 °C (23.75 ± 0.96 individuals), increased to 26.75 ± 0.50 individuals at 30 °C, and slightly declined at 35 °C (24 ± 3.16 individuals). The differences in fecundity among the treatments were not statistically significant (*p* > 0.05) (Figure 2E). The lifespan of *B. plicatilis* decreased significantly with increasing temperature, from 270.62 ± 30.38 h at 20 °C to 71.00 ± 12.73 h at 35 °C (*p* < 0.001). These results highlight the significant influence of temperature on lifespan, with higher temperatures resulting in shorter lifespans (Figure 2F).

### 3.3. Effects of Diet on B. plicatilis

**Figure 3:**
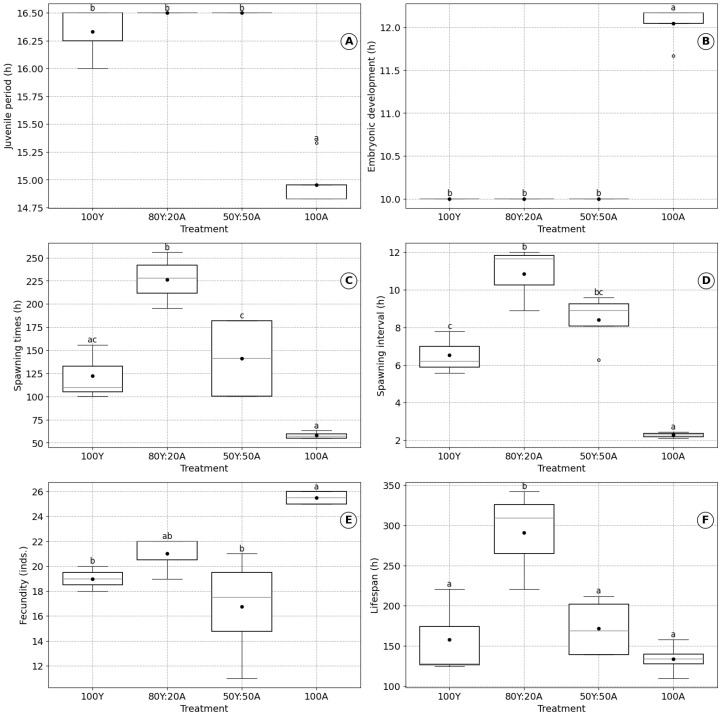
Effect of diet on the biological characteristics of *B. plicatilis*: (**A**) juvenile period, (**B**) embryonic development time, (**C**) spawning times, (**D**) spawning interval, (**E**) fecundity, and (**F**) lifespan. Data are presented as mean ± standard error based on four replicates. Different lowercase letters above boxplots indicate significant differences among treatments ac-cording to Tukey’s HSD post-hoc test (*p* < 0.05).

The experimental results demonstrated that dietary treatments had a significant influence on the biological characteristics of *B. plicatilis*, as shown in Figure 3. As mentioned above, the feeding regimes tested for rotifers included 100% baker’s yeast (100Y), an 80% baker’s yeast and 20% *Chlorella* mixture (80Y:20A), a 50% baker’s yeast and 50% *Chlorella* mixture (50Y:50A), and 100% *Chlorella* (100A). 

The juvenile period of *B. plicatilis* was shortest when fed exclusively on *C. vulgaris* algae (100A: 14.96 ± 0.25 h) and longest when fed yeast-based diets or mixtures (16.50 ± 0.00 h for 100Y, 80Y:20A, and 50Y:50A). The differences in the juvenile period between 100A and yeast-containing diets were statistically significant (*p* < 0.001), indicating that feeding entirely on *C. vulgaris* algae optimizes the juvenile period (Figure 3A). Conversely, the embryonic development time was shortest at 100Y or in mixed diets (80Y:20A and 50Y:50A, 10.00 ± 0.00 h each) and longest at 100A (12.04 ± 0.25 h). These differences were highly significant (*p* < 0.001), suggesting that yeast-based diets reduce embryonic development time compared with the *C. vulgaris* algae alone diet (Figure 3B).

The spawning times were longest in the 80Y:20A treatment (226.50 ± 30.28 h) and shortest with 100A (58.04 ± 3.79 h). Intermediate values were observed for 100Y (119.00 ± 32.04 h) and 50Y:50A (141.25 ± 47.05 h). The differences between diets were statistically significant (*p* < 0.001), indicating that mixed diets prolong spawning times (Figure 3C). The spawning interval was shortest with 100A (2.28 ± 0.14 h) and progressively increased with yeast-based diets, being longest in the 80Y:20A treatment (10.84 ± 1.70 h). The significant differences among the treatments (*p* < 0.001) indicate that a diet of *C. vulgaris* algae alone provides the most stable and shortest spawning intervals (Figure 3D).

Fecundity was highest in the 100A treatment (25.50 ± 0.58 individuals) and declined with increasing yeast proportions, with 80Y:20A (21 ± 1.73 individuals) exhibiting higher fecundity than 100Y and 50Y:50A (18.67 ± 1.15 individuals and 16.75 ± 4.35 individuals, respectively). The differences in fecundity among the diets were statistically significant (*p* < 0.01) (Figure 3E). The lifespan of *B. plicatilis* was longest at 80Y:20A (290.50 ± 62.83 h) and reduced in 50Y:50A (172.13 ± 38.64 h), 100Y (156.83 ± 55.14 h), and 100A (134.00 ± 19.60 h). The lifespan differences among the treatments were significant (*p* < 0.01), indicating that mixed diets prolong the lifespan, while single-source diets (algae or yeast alone) reduce it (Figure 3F).

**Figure 4:**
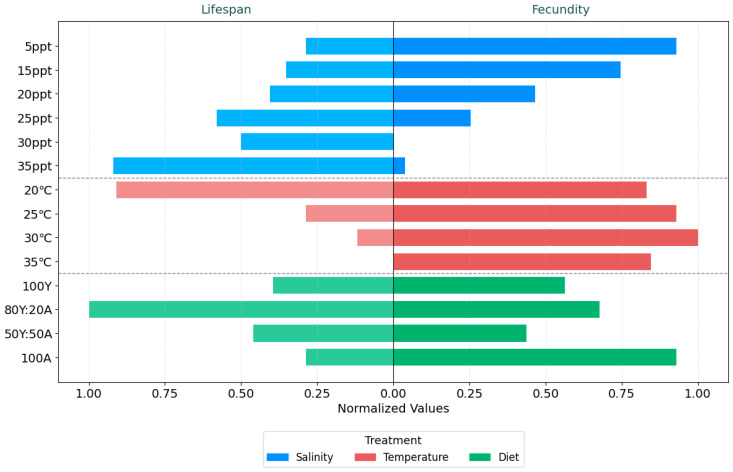
Trade offs between fecundity and lifespan in *B. plicatilis* under varying environmental conditions.

Following the analysis of the individual effects of temperature, salinity, and diet on the biological characteristics of *B. plicatilis*, an integrated visualization was constructed to illustrate the relative trade offs between lifespan and fecundity across all tested conditions (Figure 4). The values were normalized against the highest recorded values within each trait group, allowing for a direct comparison of trade off patterns under each condition.

In salinity treatments (blue bars), *B. plicatilis* displayed a consistent trade off between lifespan and fecundity. As salinity increased, lifespan extended gradually, from 134.00 h at 5 ppt to 273.00 h at 35 ppt, while fecundity showed a declining trend, dropping from 25.50 to just 9.67 individuals. The most pronounced contrast occurred between these two extremes, where the individuals lived significantly longer but reproduced at a markedly lower rate. In the temperature treatments (red bars), an opposite pattern emerged. Lifespan shortened as temperature increased, falling from 270.63 h at 20 °C to 71.00 h at 35 °C. Meanwhile, fecundity generally rose, peaking at 30 °C with 26.75 individuals. Although a slight drop in fecundity was observed at 35 °C (in 24 individuals), the values remained relatively high across this temperature range. The dietary treatments (green bars) revealed more variation. The 80Y:20A combination supported the longest lifespan (290.50 h) along with relatively high fecundity (21 individuals). In contrast, the 100A group exhibited one of the shortest lifespans (134.00 h) but maintained high reproductive output (25.50 individuals). The 50Y:50A diet resulted in intermediate values for both traits (172.13 h and 16.75 individuals), without extreme shifts in either direction.

These results reveal distinct patterns of life history modulation by the cultural factors. Salinity induced a strongly antagonistic relationship between the two traits, promoting longevity while suppressing fecundity. Temperature was found to regulate a relatively linear trade off between lifespan and reproductive output. Diet, on the other hand, exerted nonlinear effects, with specific combinations enhancing or impairing both traits in a highly condition-dependent manner. These differential responses underscore the complexity of cultural influence on rotifer biology and the importance of multifactorial analyses in understanding organismal performance.

## 4. Discussion

Cultural factors play a pivotal role in shaping the biological performance of aquatic organisms, with rotifers presenting a particularly intriguing model for understanding adaptive strategies in dynamic ecosystems. The research across various ecological contexts has consistently demonstrated the sensitivity of aquatic microorganisms to environmental parameters, emphasizing the need to understand species-specific responses [21,22,23,24]. Building on this foundational ecological research, our experimental investigation of *B. plicatilis* reveals nuanced interactions between specific environmental parameters and biological performance. Through a systematic examination, a comprehensive summary of these physiological responses under defined environmental gradients has been produced, outlining how salinity, temperature, and nutritional conditions modulate energy allocation, life history strategies, and overall biological performance in *B. plicatilis* (Table 1). To gain deeper insights into the observed biological trends, we analyzed them using established ecological theoretical frameworks, integrating the dynamic energy budget (DEB) framework and r/K selection theory to elucidate the complex energy allocation strategies of *B. plicatilis* [25,26,27,28,29,30]. The DEB framework was systematically applied to illuminate the hierarchical partitioning of finite energy resources across fundamental biological processes [31,32]. Energy flow was meticulously tracked from nutritional intake through metabolic maintenance, growth, and reproductive outputs [33]. Consequently, hierarchical energy allocation was observed to prioritize metabolic maintenance mechanisms before investment in growth and reproductive processes [34,35]. In the specific context of *B. plicatilis*, a sophisticated energy redistribution mechanism was identified, wherein organismal energy allocation was dynamically modulated in response to environmental stressors, enabling strategic reallocation between survival and reproductive strategies [36]. Complementary insights were derived from r/K selection theory, which provided a comprehensive explanation of the diverse reproductive strategies that exist along a continuum between r-selected traits (characterized by rapid reproduction, high fecundity, and abbreviated lifespans) and K-selected traits (distinguished by measured reproduction, extended longevity, and enhanced survival investment) [21,37].

### 4.1. Effects of Salinity on Physiological Mechanisms

Salinity constitutes a pivotal environmental factor that imposes complex physiological demands on *B. plicatilis*, initiating a series of coordinated molecular and cellular adaptive responses [38]. The species’ ability to endure a broad salinity spectrum is underpinned by a robust oxidative stress management system that significantly influences its life history trajectory [39]. This adaptability is reflected in empirical observations. At 5 ppt, *B. plicatilis* exhibits optimal reproductive performance, with a juvenile period of 14.96 ± 0.25 h, embryonic development of 12.04 ± 0.25 h, and fecundity reaching 25.50 ± 0.58 individuals (Figure 1A,B,E). However, under high-salinity conditions (35 ppt), substantial physiological shifts are evident, as indicated by the extension of the juvenile period to 67.33 ± 5.77 h, prolonged embryonic development to 22.00 ± 0.87 h, and a notable reduction in fecundity to 9.67 ± 5.03 individuals (Figure 1A,B,E).

At the molecular level, the exposure to elevated salinity (25–35 ppt) results in increased intracellular reactive oxygen species (ROS), activating a dynamic antioxidant defense system [40]. This response involves a tightly regulated enzymatic mechanism that mitigates oxidative damage, functioning not merely as a passive defense but as an active strategy for physiological adjustment under osmotic stress. These molecular adaptations are accompanied by shifts in energy allocation that align with r/K selection theory [41,42]. At lower salinities, such as 5 ppt, *B. plicatilis* adopts an r-selected strategy, characterized by rapid development and high fecundity. In contrast, under high-salinity conditions (25–35 ppt), the species transitions towards a K-selected strategy, favoring an extended lifespan and a reduced reproductive output.

Despite delayed population growth and reduced reproductive success at extreme salinities, *B. plicatilis* exhibits remarkable phenotypic plasticity [43]. The maximum recorded lifespan of 273.00 ± 72.52 h at 35 ppt illustrates a metabolic reconfiguration oriented towards survival, in which energy is diverted from reproduction to somatic maintenance. This adjustment ensures persistence under environmentally challenging conditions [44,45,46].

Our findings are consistent with the substantial body of literature that demonstrates the significant impact of salinity on rotifer life history traits. Supporting these findings, previous studies have consistently reported the detrimental effects of salinity fluctuations on rotifer performance. In *B. plicatilis*, complete mortality was observed at both 0 and 40 ppt, with reproductive output peaking at lower salinities (5–10 ppt) and decreasing significantly at 20–35 ppt [47]. Similarly, *B. koreanus* displayed a marked reduction in cumulative offspring production at 25 and 35 ppt [48,49], and in *Synchaeta littoralis*, fecundity was lower at 30 and 35 ppt compared with 25 ppt [50]. Collectively, these results underscore the influence of salinity on rotifer physiological mechanisms and highlight the importance of species-specific salinity optimization for enhancing reproductive efficiency and sustaining ecological performance under variable environmental conditions.

### 4.2. Effects of Temperature on Physiological Mechanisms

The remarkable stability of fecundity in *B. plicatilis* across varying temperatures provides compelling evidence for metabolic acceleration as a primary physiological response rather than environmental stress (Figure 4). At the molecular level, this adaptive capacity is mediated through a sophisticated network of cellular mechanisms, primarily orchestrated by heat shock proteins (HSPs) and antioxidant defense systems that transform temperature variations from potential stressors into signals for metabolic optimization [51]. As temperatures increase from 20 °C to 30 °C, the species demonstrates a capacity for metabolic optimization, with egg production remaining remarkably stable at approximately 25 eggs, despite significant changes in other physiological parameters. This stability is underpinned by a complex molecular response involving the precise regulation of gene expression, particularly heat shock proteins and antioxidant-related genes [52].

The heat shock protein system, particularly hsp40, hsp60, and hsp70, plays a crucial role in maintaining cellular proteostasis, acting as molecular chaperones that protect and refold proteins during thermal transitions [53,54]. The progressive acceleration of metabolic processes is evident in the dramatic reduction in time required for juvenile and embryonic development. At 20 °C, the juvenile period spans 33.38 ± 4.52 h, with embryonic development taking 19.62 ± 4.55 h (Figure 4). This progressively decreases to 9.00 h for the juvenile period and 8.00 h for embryonic development at 30 °C, suggesting an efficient metabolic adaptation rather than a stress response [55,56]. Simultaneously, the organism’s antioxidant defense system undergoes precise modulation. Glutathione S-transferases (GSTs), superoxide dismutase (SOD), and catalase (CAT) are dynamically regulated to manage reactive oxygen species (ROS) levels, preventing oxidative damage while supporting metabolic acceleration [57]. This is not a passive defensive mechanism but an active metabolic recalibration strategy, enabling the rotifer to perceive temperature as an informational signal rather than a threat [58].

Previous research supports this interpretation of thermal-induced metabolic optimization. Studies on the *Brachionus* species have demonstrated that temperature modulates metabolic rates without compromising reproductive potential, a characteristic that aligns with the dynamic energy budget (DEB) theoretical frameworks [59]. This ability to maintain consistent fecundity while accelerating internal processes reflects an evolutionary adaptation to thermal variability. Only at 35 °C does a critical threshold emerge, where all physiological parameters simultaneously decline. This suggests a specific thermal limit rather than a generalized stress response, indicating that *B. plicatilis* possesses a remarkable range of metabolic plasticity [60]. The consistent egg production across temperatures, varying only between 23.75 ± 0.96 and 26.75 ± 0.50 eggs, provides direct evidence of the organism’s ability to maintain reproductive output despite the changing metabolic conditions. From an evolutionary perspective, this metabolic strategy aligns with r/K selection theory, demonstrating how aquatic microorganisms can dynamically reallocate energy resources to optimize survival and reproduction under varying environmental conditions [61,62]. The findings challenge simplistic environmental stress models, instead revealing temperature as a nuanced metabolic regulator that drives physiological optimization. Comparative studies on *Euchlanis dilatata* and other *Brachionus* species further substantiate this interpretation, showing similar patterns of metabolic acceleration and reproductive stability across thermal gradients [37]. This suggests a broader adaptive mechanism among aquatic microorganisms that enables efficient energy reallocation in response to environmental temperature fluctuations.

### 4.3. Effects of Diet on Physiological Mechanisms

Diet plays a pivotal role in determining the biological characteristics of *B. plicatilis*. Our results demonstrated that feeding on 100% *C. vulgaris* algae resulted in the highest fecundity (25.50 ± 0.58 inds.) but was associated with a shorter lifespan (134.00 ± 19.60 h) (Figure 4). This supports the findings of Sun et al. (2017), who also observed reduced lifespans in rotifers fed exclusively on *C. vulgaris* [63]. Conversely, the diets incorporating yeast or a mixture of yeast and algae extended lifespan, with the longest lifespan observed in the 80Y:20A treatment (290.50 ± 62.83 h). This trend aligns with the findings of Sun et al. (2017) [63], which indicated that the lifespan of *B. plicatilis* was extended when it was fed a mixed diet consisting of 50% *C. vulgaris* and 50% *P. globosa* [22]. Furthermore, the embryonic development time was also shorter when yeast-based diets were used, suggesting that yeast may accelerate metabolic processes. However, fecundity decreased when yeast was included in the diet, reflecting a trade off between longevity and reproductive output. These findings emphasize the importance of diet composition in optimizing rotifer cultures, with mixed diets offering a balance between extended lifespan and moderate fecundity. 

Nutrient-dense diets, particularly those high in essential fatty acids and proteins, can enhance fecundity but may reduce lifespan. For instance, studies on *B. plicatilis* reveal that those fed protein-rich algae, such as *Isochrysis galbana,* produced more offspring, yet had shorter lifespans. Mechanistically, the trade off between reproduction and lifespan can be explained through oxidative stress and nutrient signaling pathways. According to [30,64], diets that promote reproduction often increase oxidative damage due to heightened metabolic activity, resulting in shorter lifespans. In contrast, energy-efficient diets reduce oxidative stress, enabling longer survival but at the expense of reduced fecundity. This balance is further modulated by environmental stressors, such as salinity and temperature, which affect metabolic rates, osmoregulatory demands, and the biochemical composition of rotifers.

This balance between survival and reproduction can be further understood within the r/K selection framework. Rotifers, as opportunistic organisms, exhibit r-selected traits in unstable environments, favoring rapid reproduction, short lifespans, and high fecundity to maximize fitness before conditions become unfavorable. For instance, studies on *B. plicatilis* and *B. manjavacas* demonstrate that these species adjust their reproductive strategies in response to environmental fluctuations, with higher salinity and temperature often accelerating reproductive rates but shortening lifespan [36]. Conversely, under more stable conditions, K-selected traits emerge, such as slower reproduction, longer lifespans, and increased energy investment in diapause eggs, ensuring persistence through adverse periods [36]. Moreover, the trade offs between egg size and reproductive output are a key determinant of rotifer life history strategies. For example, species like *Euchlanis dilatata* produce larger eggs at lower temperatures, favoring offspring survival over immediate reproductive success [30]. In contrast, *B. plicatilis* populations experiencing food limitation shift their strategy by producing fewer but higher quality eggs or investing in diapause, ensuring long-term persistence [56]. Such trade offs highlight phenotypic plasticity in energy allocation, where reproductive effort is modulated in response to resource availability and environmental predictability. Our findings align with this dynamic, highlighting how specific environmental conditions (e.g., salinity, temperature, and diet) influence the strategic trade off between reproduction and survival in *B. plicatilis.* Understanding these trade offs is essential for optimizing rotifer culture systems in aquaculture, where specific traits (e.g., high fecundity for rapid population growth or extended lifespan for sustained culture) can be targeted based on environmental and nutritional conditions. Further research into the molecular and physiological mechanisms underlying these trade offs will provide deeper insights into the adaptability and resilience of rotifers under varying conditions.

The influence of various environmental factors, such as temperature, salinity, and diet, on metabolic processes, cellular responses, gene expression, and physiological outcomes in aquatic microorganisms is summarized in Table 2. The table indicates that temperature increases metabolic rates and enhances energy allocation to growth and reproduction at moderate levels, whereas high temperatures lead to faster development but shorter lifespans. Salinity, on the other hand, triggers osmotic regulation mechanisms and shifts energy allocation from reproduction to survival at high salinity, with the corresponding upregulation of stress-related genes. Diet plays a significant role in growth efficiency, with *Chlorella-*based diets enhancing protein synthesis and growth, while yeast-based diets extend lifespan by redistributing energy. Additionally, dietary composition influences gene expression, with growth-related genes being upregulated with algae diets and reproductive genes being downregulated in yeast-rich diets.

## 5. Conclusions

This study highlights the significant influence of salinity, temperature, and diet on the biological characteristics of *B. plicatilis*, providing valuable insights for aquaculture optimization. Specifically, at 5 ppt salinity, *B. plicatilis* achieved the highest fecundity (25.50 ± 0.58 individuals), while the longest lifespan (273.00 ± 72.52 h) was observed at 35 ppt, indicating salinity-dependent energy allocation. Similarly, the temperature profoundly affected biological traits, with rapid juvenile development at 35 °C (8.00 ± 0.00 h) and extended lifespan at 20 °C (270.62 ± 30.38 h). Furthermore, diet quality was crucial, with *C. vulgaris* supporting maximum fecundity, whereas mixed diets, such as 80% yeast and 20% *C. vulgaris*, extended lifespan (290.50 ± 62.83 h), highlighting a trade off between reproduction and longevity. These findings highlight the importance of precise environmental management in tailoring rotifer cultures to meet specific aquaculture needs, thereby balancing reproduction and survival. Future research should focus on the underlying molecular mechanisms to refine strategies for maximizing productivity and sustainability in rotifer-based systems.

## Figures and Tables

**Table 1:** Environmental factors and their impact on biological performance and r/K selection strategies.

Environmental Factor	Sub-Optimal Condition	Optimal Condition	Adverse Condition	References
Salinity	Low salinity (5 ppt)	Moderate salinity (10–20 ppt)	High salinity (25–35 ppt)	Figure 1
Temperature	High temperature (30–35 °C)	Moderate temperature (20–25 °C)	Low temperature (<20 °C)	Figure 2
Nutritional Conditions	80% baker’s yeast–20% *C. vulgaris* (80Y:20A)	50% baker’s yeast–50% *C. vulgaris* (50Y:50A)	100% *C. vulgaris* (100A)	Figure 3
DEB Strategy	Optimal energy uptake → high reproductive potential	Balanced energy allocation	Energy redirection to survival	[32]
r/K selection	r-selection dominance	Intermediate r/K strategy	K selection prominence	[27,31]
Biological Performance	Accelerated reproduction	Balanced growth and reproduction	Reduced reproduction, extended survival	[22]

**Table 2:** Summary of the effects of environmental factors on metabolic, cellular, and physiological responses in rotifers.

Environmental Factor	Metabolic Processes	Cellular Responses	Gene Expression	Physiological Outcomes	References
Salinity	- Osmotic regulation mechanisms are activated - Shift in energy allocation from reproduction to survival at high salinity	- Ion transport adjustments via Na+/K+ pumps - Increased antioxidant activity at extreme salinity levels	- Upregulation of SOD, CAT, HSPs at high salinity - Enhanced expression of stress response genes	- High fecundity at low salinity (5–15 ppt) - Extended lifespan at high salinity (25–35 ppt) due to stress adaptation	[39,65,66] [22,67] [15,45]
Temperature	- Increased metabolic rate at higher temperatures - Enhanced energy allocation to growth and reproduction at moderate temperatures	- Heat shock protein (HSP) activation - Antioxidant enzyme regulation (SOD, CAT, GST)	- Upregulation of hsp40, hsp60, hsp70 - Enhanced expression of antioxidant-related genes	- Faster development at 30–35 °C - Shorter lifespan at high temperatures - Increased fecundity at optimal temperatures	[21,23,68] [69] [10,30]
Diet	- High protein synthesis with *Chlorella*-based diets - Energy redistribution with yeast-based diets	- Improved growth efficiency with *Chlorella* - Extended lifespan with yeast-rich diets	- Upregulation of growth-related genes with algae diets - Downregulation of reproductive genes in yeast-dominant diets	- Maximum fecundity with 100A - Longer lifespan with 80Y:20A - Faster embryonic development in yeast-rich diets	[70,71] [14] [72,73]

## Data Availability

The raw data supporting the conclusions of this article will be made available by the authors on request.
